# Novel targets for potential therapeutic use in Diabetes mellitus

**DOI:** 10.1186/s13098-023-00983-5

**Published:** 2023-02-13

**Authors:** Sanchit Dhankhar, Samrat Chauhan, Dinesh Kumar Mehta, Nitika, Kamal Saini, Monika Saini, Rina Das, Sumeet Gupta, Vinod Gautam

**Affiliations:** 1 Department of Pharmaceutical Sciences, M.M. College of Pharmacy, Maharishi Markandeshwar (Deemed To Be University), Mullana, Ambala, 133207 Haryana India; 2 Department of Pharmaceutical Sciences, IES Institute of Pharmacy, IES University, Bhopal, India; 3 Chitkara College of Pharmacy, Chitkara University, Rajpura, 140401 Punjab India; 4 Ganpati Institute of Pharmacy, Bilaspur, Yamunanagar, 135102 Haryana India

**Keywords:** Diabetes mellitus, New drug molecules, Novel targets, Unexplored targets

## Abstract

Future targets are a promising prospect to overcome the limitation of conventional and current approaches by providing secure and effective treatment without compromising patient compliance. Diabetes mellitus is a fast-growing problem that has been raised worldwide, from 4% to 6.4% (around 285 million people) in past 30 years. This number may increase to 430 million people in the coming years if there is no better treatment or cure is available. Ageing, obesity and sedentary lifestyle are the key reasons for the worsening of this disease. It always had been a vital challenge, to explore new treatment which could safely and effectively manage diabetes mellitus without compromising patient compliance. Researchers are regularly trying to find out the permanent treatment of this chronic and life threatening disease. In this journey, there are various treatments available in market to manage diabetes mellitus such as insulin, GLP-1 agonist, biguanides, sulphonyl ureas, glinides, thiazolidinediones targeting the receptors which are discovered decade before. PPAR, GIP, FFA1, melatonin are the recent targets that already in the focus for developing new therapies in the treatment of diabetes. Inspite of numerous preclinical studies very few clinical data available due to which this process is in its initial phase. The review also focuses on the receptors like GPCR 119, GPER, Vaspin, Metrnl, Fetuin-A that have role in insulin regulation and have potential to become future targets in treatment for diabetes that may be effective and safer as compared to the conventional and current treatment approaches.

**Graphical Abstract:**

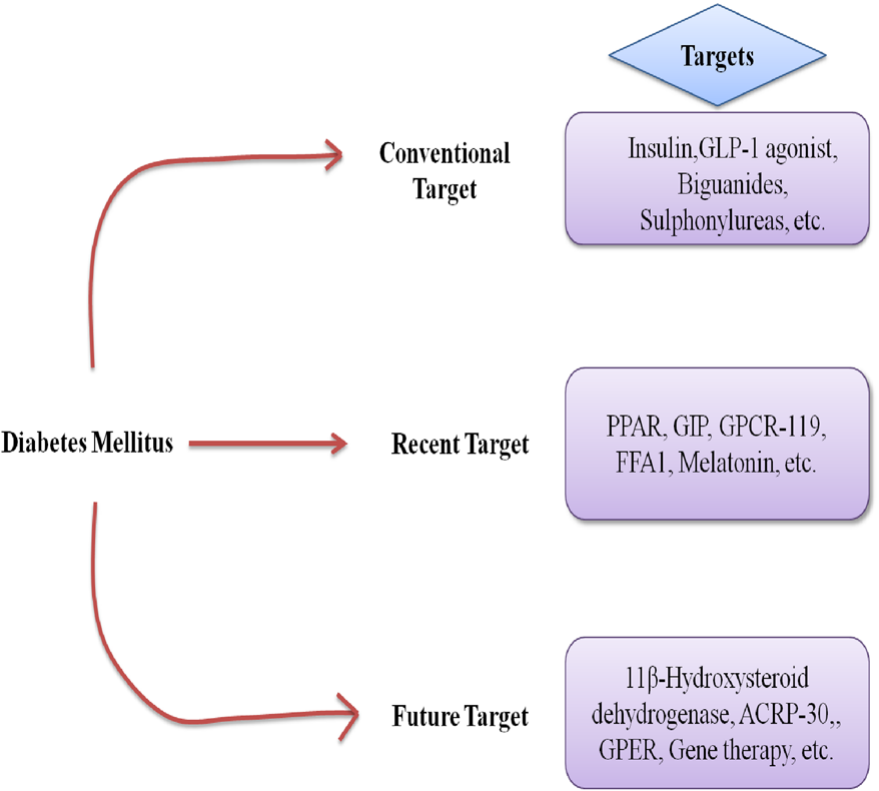

## Introduction

Diabetes mellitus (DM) is a group of metabolic illnesses characterized by a constant increase in blood sugar concentration in which the pancreas is not able to produce enough insulin from β-cells or the insulin is unable to bind to its receptors due to which there is an increase in the amount of blood glucose level [1]. Recent studies predict that the prevalence of diabetes in adults will rise from 4% in 1995 to 6.4 per cent by 2025, data was collected from recent surveys [2]. Currently DM is being treated by using anti-diabetic drugs like metformin, sulfonylurea, thiazolidinedione or DPP-4 inhibitors [3]. However, these medications are unable to control diabetes completely, and research is ongoing to develop a better treatment. Receptors are chemical structures made up of proteins that receive and transmit signals in biological systems [4]. These are some of the receptors and drugs that are now being employed in the treatment of diabetes for e.g. Insulin [5], GLP-1 [6], PPAR’s [7], Biguanides [8], Sulphonylureas [5], Glinides [5], Thiazolidinediones [5], Gliptins [5], α- Glucosidase inhibitors [5], Amylin analogues [5], SGLT-2 [9], Dopamine D-2 agonists [10].

When blood glucose levels reach high, β cells of the pancreas are participating actively and release the insulin which subsequently attaches to its receptor to activate it. Exo protease carboxypeptidase and pro-hormone convertases (PC I and PC 2) synthesize insulin from pro-insulin. These enzymes are accountable for the generation of insulin and C-peptide [11]. Insulin allows the (GLUT4) to be translocated to the cell, due to which body cells (adipose/skeletal muscle cells) consume some extra glucose. This functions in the regularization of blood glucose levels [12].

There are also other novel targets for diabetes mellitus control that could be exploited, such as GPCR 119 [13], GPER [14], 11β-hydroxysteroid dehydrogenase 1 [15], Vaspin [16], Metrnl [17], PEDF [18], Fetuin-A [19], ACRP 30(AdipoQ) [20], Visfatin, Melatonin [21], GIP [22], GPCR [23]. These targets could be the future of the diabetes treatment.

### Conventional targets in diabetes

**Fig. 1:**
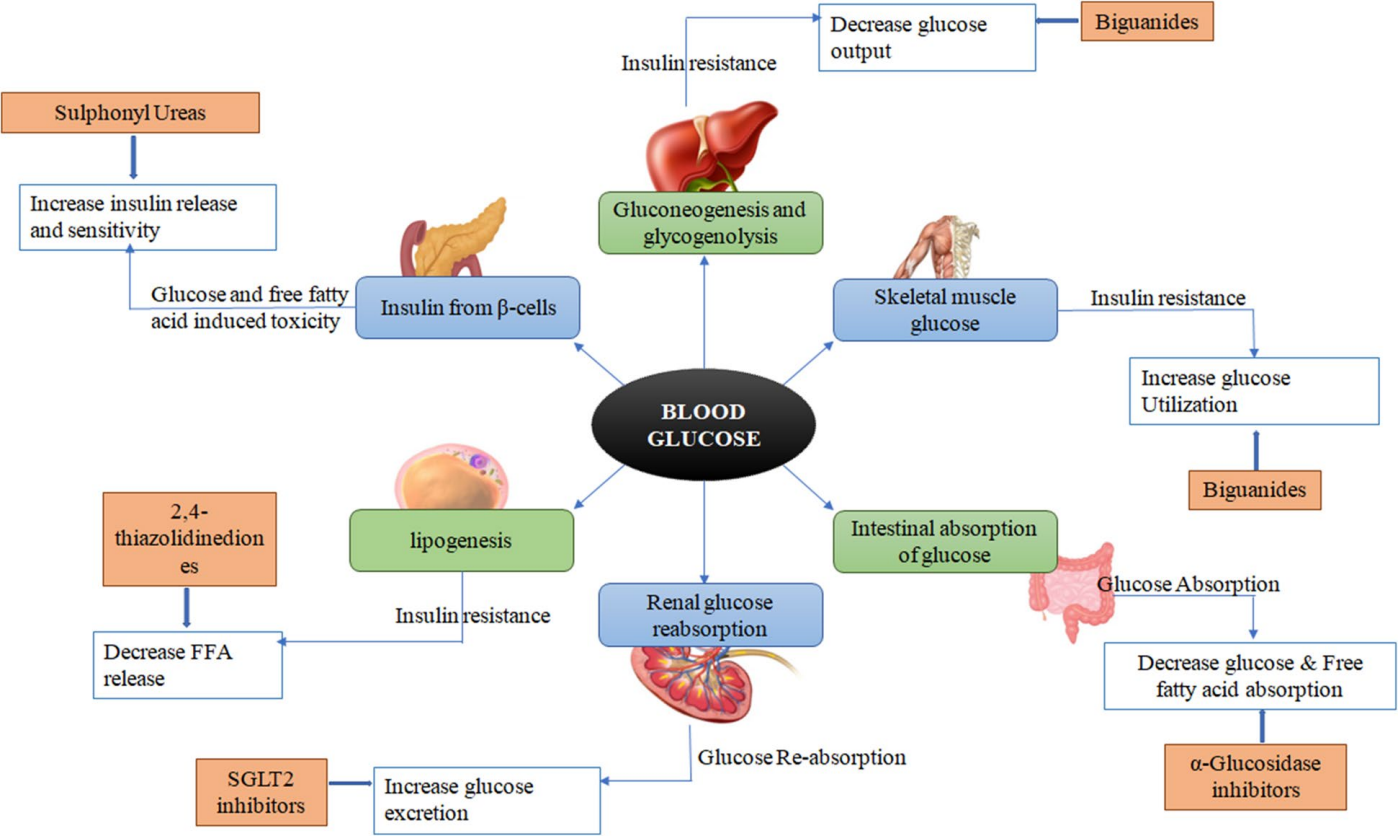
Roles of different conventional targets in diabetes mellitus

Conventional targets are the agents that are being used in the market for a long time for the treatment of diabetes but they are limited in number and have several disadvantages like weight gain, hypoglycemia, etc. also they only can manage the condition and delay the complications. They work by maintaining blood glucose levels, such as Biguanides which decreases glucose output and increases glucose utilization in skeletal muscles and liver. SGLT-2 inhibitors that increases the glucose excretion from the kidney. Α- Glucosidase inhibitors helps in decreasing the glucose and free fatty acids absorption from intestine. Sulphonyl ureas increases the insulin release and sensititvity from pancreas. 2,4- thiazolidinediones decreases the secretion of FFA from the fats cells (Fig. 1).

### Recent targets in diabetes

Recent targets are the receptors and mediators that are recently being targeted in the discovery of new agents for diabetes treatment. Lots of *in-silico*, *in-vitro*, *in-vivo* and clinical studies have been already done by targeting these receptors.

#### PPAR (Peroxisome proliferator-activated receptors)

**Fig. 2:**
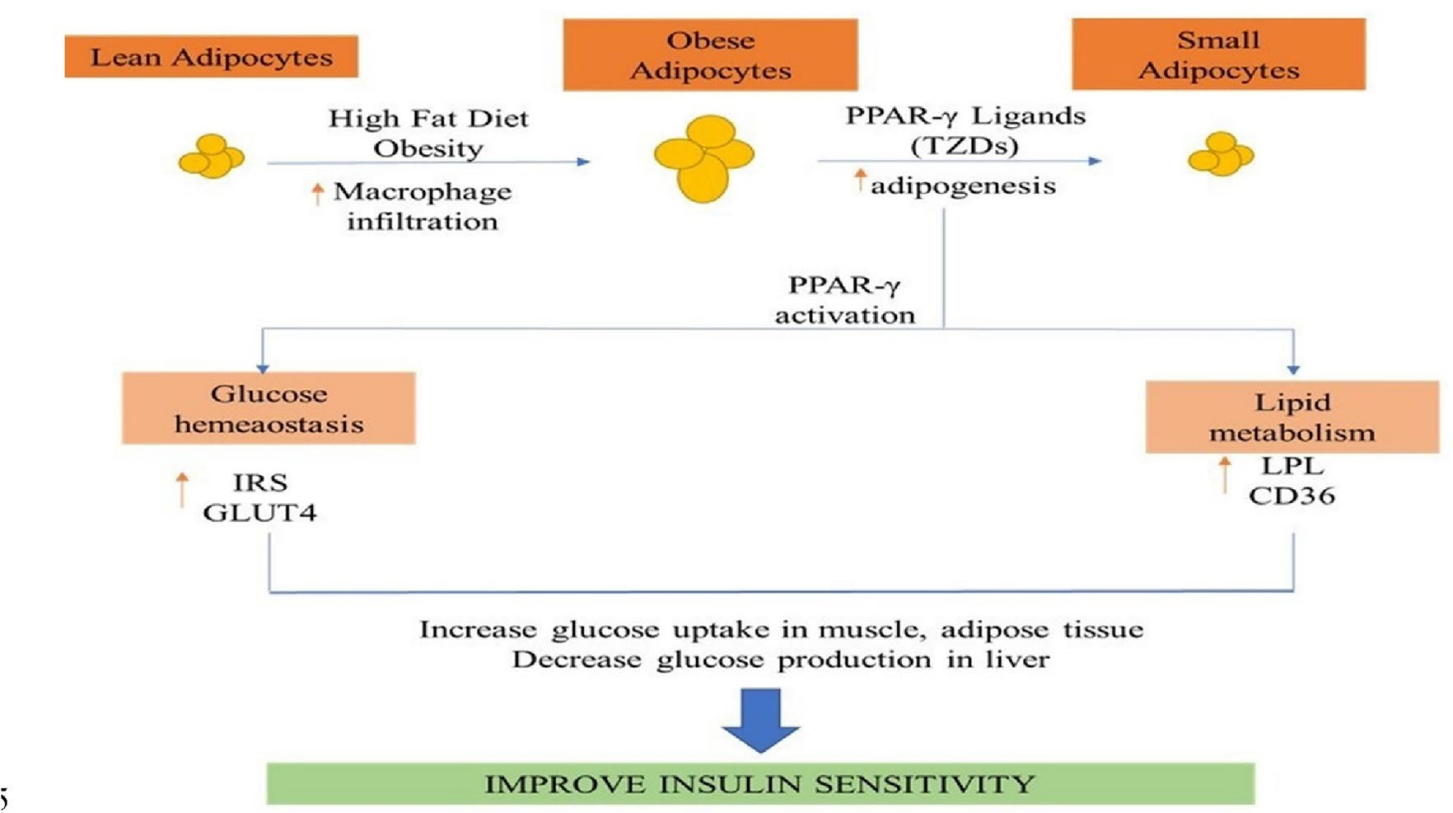
Mechanism of PPAR

Peroxisomes are the sub cellular organelles that are found in animal or human cells and play an emerging role in metabolic procedures like the metabolism of free fatty acids, cholesterol [24] and lipids to improve insulin sensitivity in the body. Peroxisome proliferator-activated receptors or PPARs functions as the transcription factors regulating the expression of genes which is divided into the three types; PPAR α, PPAR-γ, and PPARβ/δ [25]. PPAR- γ agonists (Thiazolidinediones) activates the receptor and improve overall insulin sensitivity in the body. After activating, they reduce free fatty acid levels in the blood also changes the adipokines levels, which is facilitated by lowering glucose synthesis in the liver, improving glucose intake in skeletal muscle & adipose tissues, & increasing insulin release from the pancreas (Fig. 2) [26].

#### GIP (Glucose-dependent insulinotropic polypeptide)

**Fig. 3:**
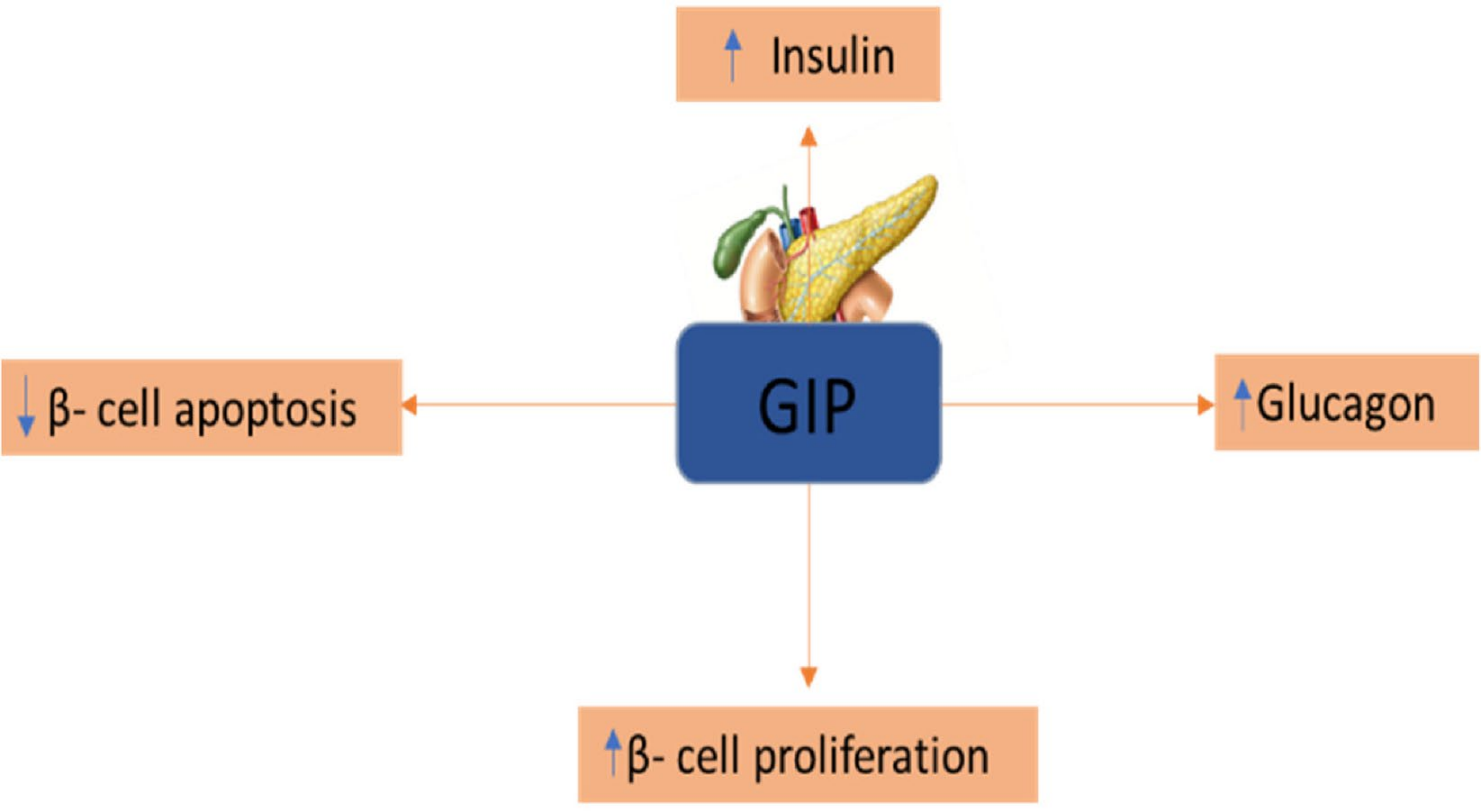
Role of GIP in diabetes

GIP is one of the incretin hormones, located in the β-cells, adipose tissue & in brain [27] where it plays an important role in the type-2 diabetes mellitus and other metabolic disorders (Fig. 3) [28, 29] by boosting the insulin response which is triggered by the post-prandial rise in glycemia [28].

### Mechanism

**Fig. 4:**
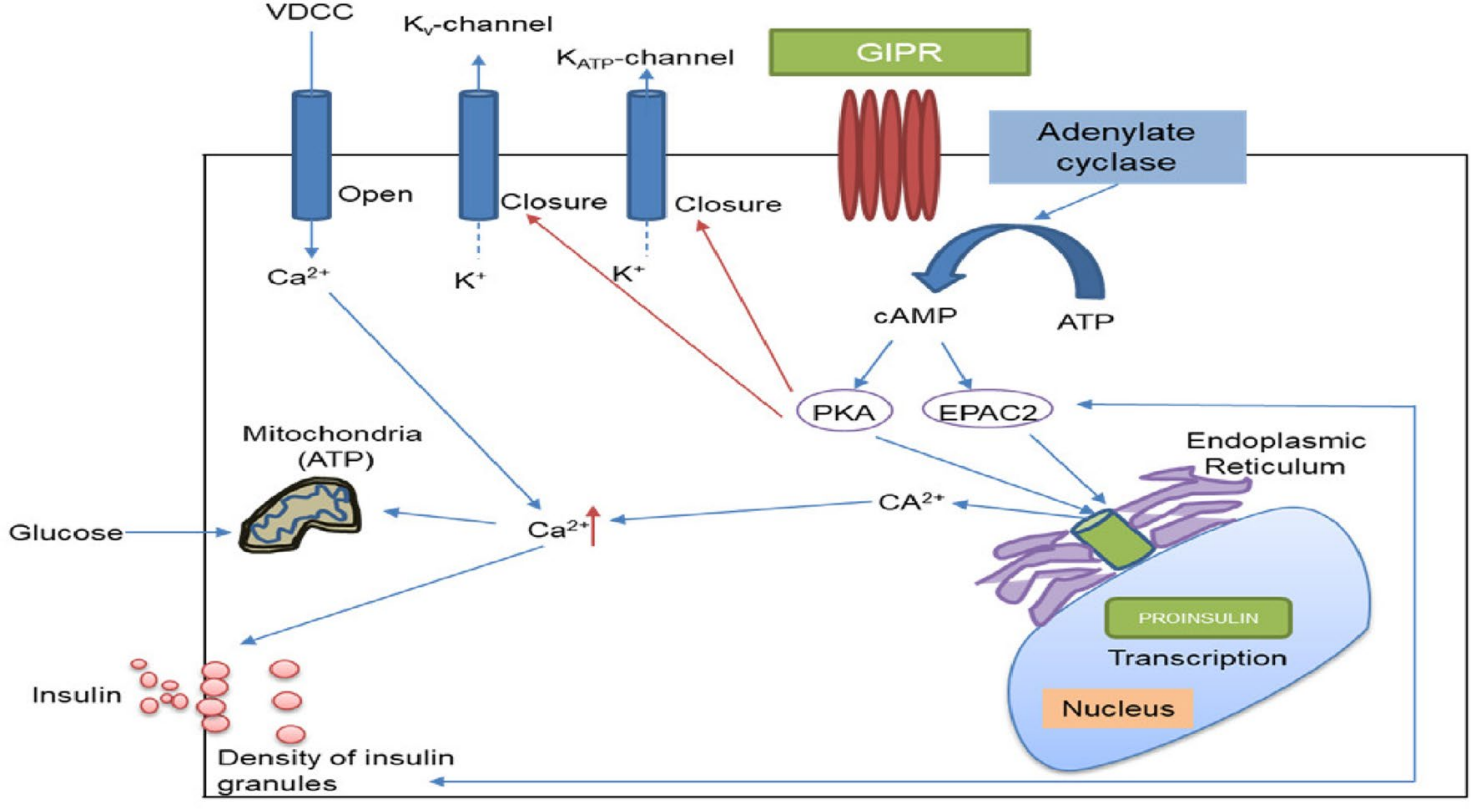
GIP mediated mechanism for insulin secreation from β-cells

GIP performs its insulinotropic action by attaching to the GIP receptor (GIPR) which increases the intracellular (cAMP) levels. Increased levels of cAMP activate the Protein kinase-A (PKA) & exchange protein activated cAMP2 (EPAC2). Depolarization of the voltage-gated Ca^+^ channels allows the rise of intracellular Ca^2+^ concentration that activates the Ca^2+^ from intracellular stores by PKA and EPAC2 mechanisms. The increase in Ca^2+^ concentration promotes the transcription of the proinsulin gene, thus help in rising the insulin secretion from β-cells (Fig. 4) [30].

#### G-Protein coupled receptor (GPCR 119)

**Fig. 5:**
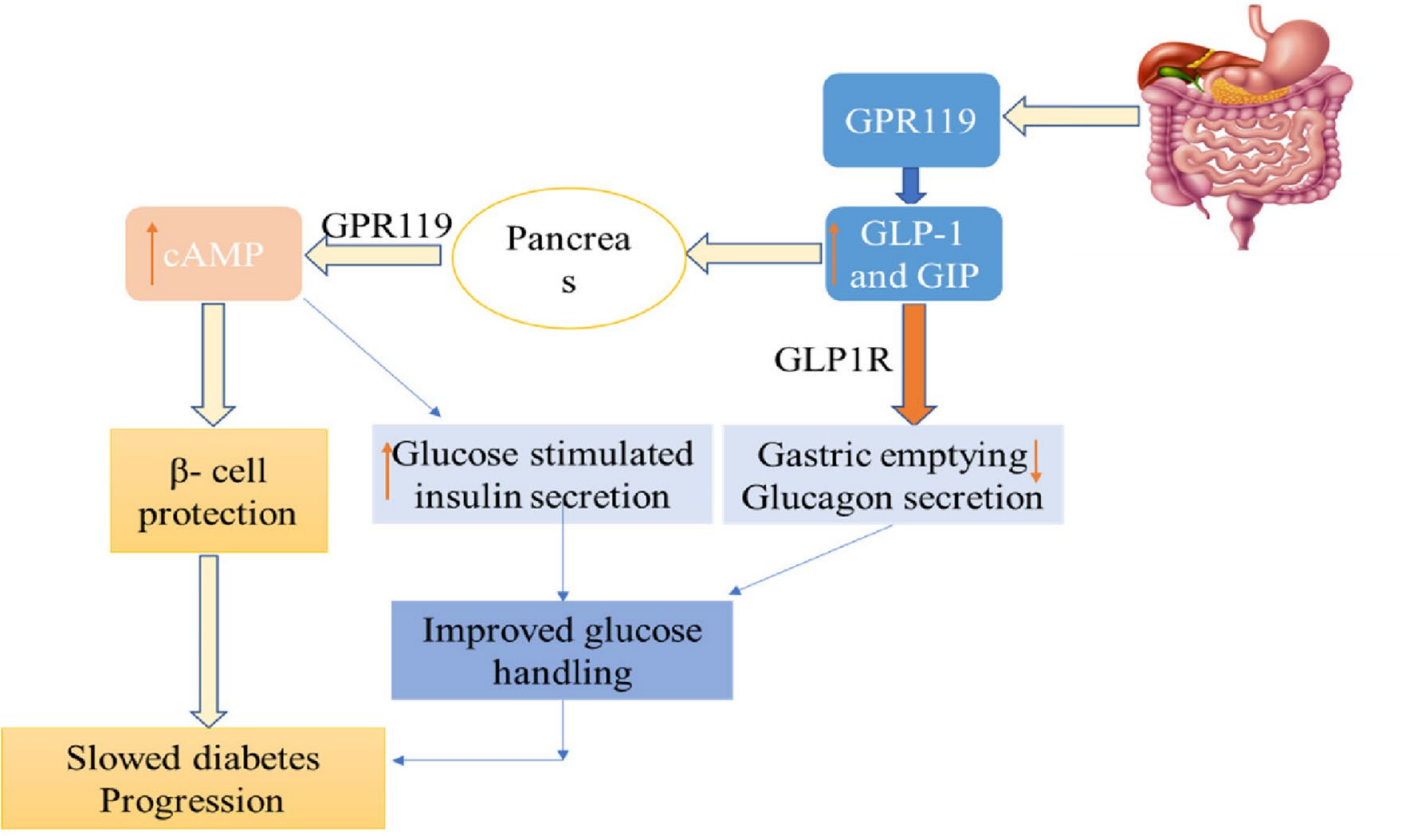
Mechanism of GPR119

GPR119 is a G-protein coupled receptor of Class-I [31], found in the muscles, liver [32] along with the β-cells of the pancreas [33]. The activation of GPR119 may similarly enhance insulin production just like incretin hormones [34, 35] and show the positive effects in insulin secretion when the agonists attached to its binding site [36, 37]. GPR119 acts in two different ways to improve glucose homeostasis, one is the direct effect on glucose-activated insulin release in β-cells & an indirect effect on the release of GLP-1 and GIP in enteroendocrine cells (Fig. 5) [38–40].

#### FFA 1 (Free fatty receptor-1)

**Fig. 6:**
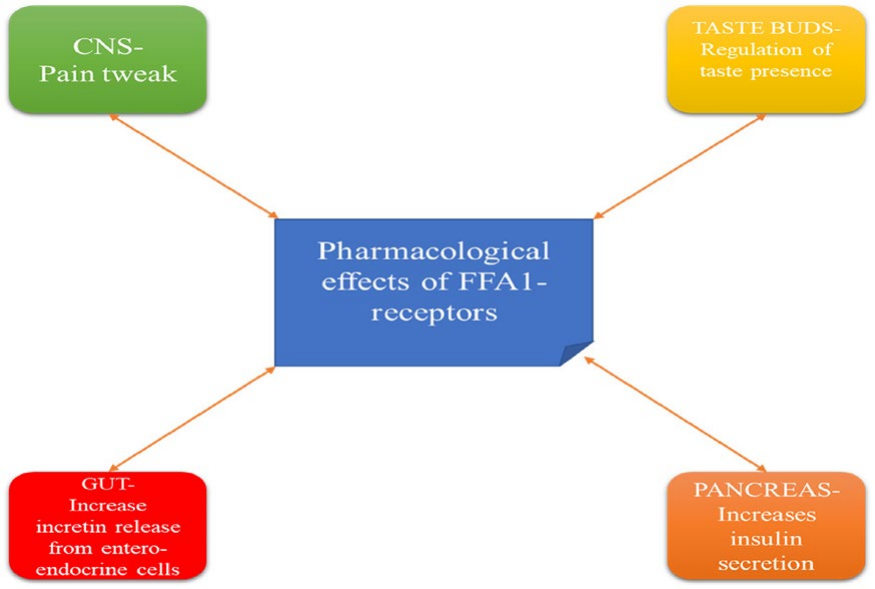
Pharmacological effects of FFA-1 receptors

FFA1 are the receptors that belong to the Class-A G-protein coupled receptor, also known as G- protein coupled receptor-40 [41]. Basically, FFA1 (Table 1) are found in the pancreatic cells, intestinal cells also found in the taste buds and central nervous system cells in mammals (Fig. 6) [42].

**Table 1:** Types of fatty acids

Types	Characteristics	References
Short-chain fatty acids (SCFAs)	1–6 carbon atoms	[46]
Medium-chain fatty acids (MCFAs)	7–12 carbon atoms	[46]
Long-chain fatty acids (LCFAs)	12 carbon atoms	[46]

**Fig. 7:**
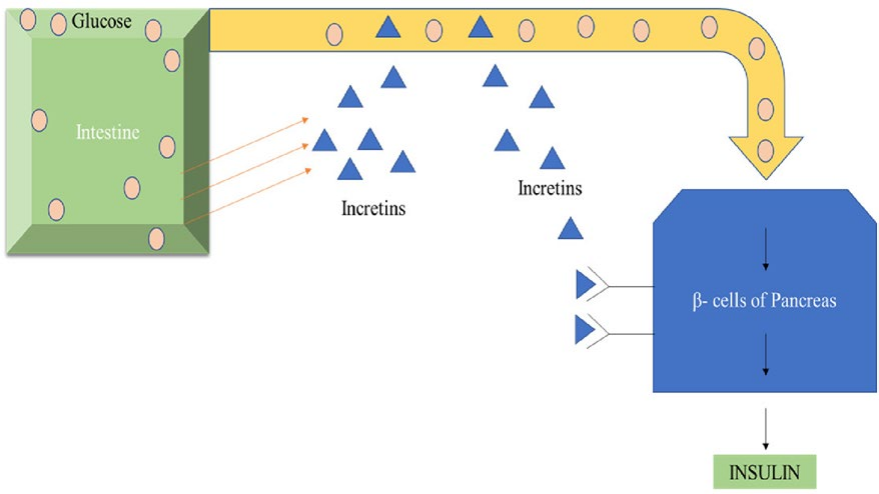
Incretin release is stimulated by the glucose present in the small intestine, then incretins are passed to their target tissue is the pancreas, to stimulate the β- cells lead them to release additional insulin in action to the equal volume of blood glucose [47]

In an *ex-vivo* study, using beta-cell lines of mouse islets, it is found that the FFA1 receptor affects the lipid and glucose metabolism [43] and increases the insulin secretion from beta-cell of the pancreas [44]. FFA1 affects the blood glucose level by two pathways: By Indirectly increasing incretin hormones as well as directly promoting insulin release from pancreatic β-cells (Fig. 7) [44, 45].

**Fig. 8:**
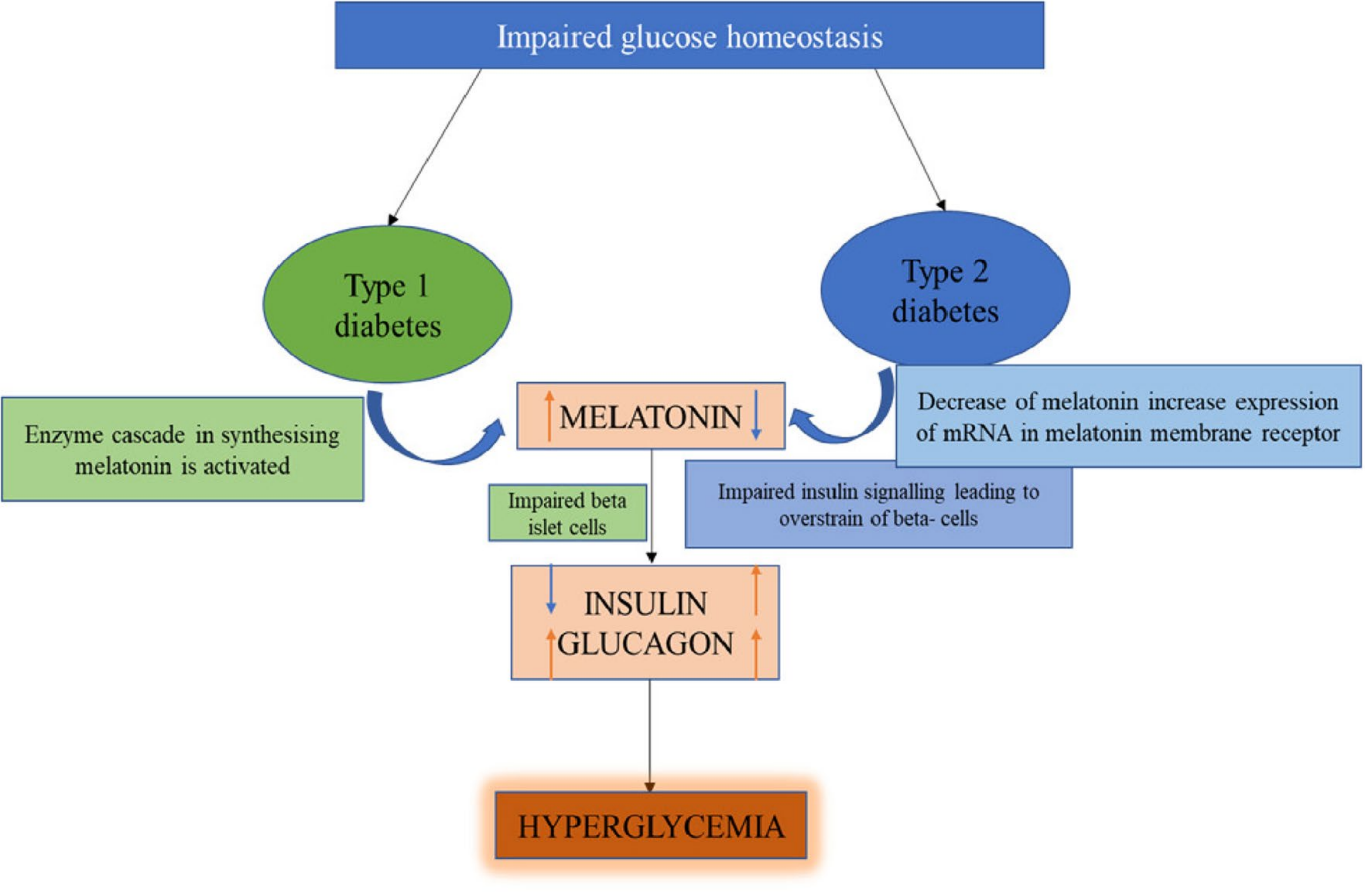
There is an increased production of Melatonin in T1DM (Type-1 diabetes mellitus) due to the activation of enzyme cascade which causes reduced β- cell function which then reduces the formation of insulin and rises the amount of glucagon in cells resulting in high blood glucose. Then in T2DM, the decreased production of Melatonin causes the increase in mRNA expression of melatonin membrane receptor which leads to the impaired insulin signaling that causes a upsurge in the insulin level leading to beta-cell exhaustion with high glucagon concentration leading to hyperglycemia

#### Melatonin

Melatonin is a neuroendocrine hormone, released from the pineal gland at night [48]. It is found that the melatonin is also responsible for glucose regulation and insulin release from pancreas [49, 50] which turns it into a possible goal for the management of the diabetes mellitus. It performs its pharmacological actions by interacting with the melatonin receptors MT1 and MT2 [50] that are present at the extracellular membrane present in several cells throughout the body [51]. It is found in the recent studies that melatonin MT1 receptor knockout-mouse [52] has shown the results with increased insulin resistance and glucose tolerance [52, 53] in them which makes the MT1 receptor of melatonin an essential target for maintaining blood glucose in the body. Also it is revealed from a clinical study that administering melatonin as treatment to the diabetic patients who have low levels of melatonin in their circulation [48], can improve their glucose levels by increasing insulin secretion [48, 54]in their body (Fig. 8).

### Future targets

Future targets, as the name suggest are the potential receptors or targets that can be new site for the developing new lead compounds in the diabetes treatment. Today, there is very little information available regarding their role in diabetes but these targets have potential to play a vital role in the treatment of diabetes (Table 2).

**Table 2:** Future Targets

Compound	Class	Mode of action	Potential role in diabetes	References
11β Hydroxysteroid dehydrogenase	Glucocorticoids	High levels cause glucose intolerance	By inhibiting 11β-HSD Decrease in blood glucose levels, improved insulin sensitivity	[55]
ACRP-30	Hormone	Low levels cause insulin sensitivity	Increase in Acrp30 will increase the insulin sensitivity and decrease in blood glucose levels	[56–58]
FETUIN-A	Glycoprotein	Involved in the inflammation of the β-cells	Low levels of Fetuin-A will increase the insulin sensitivity	[59]
VISFATIN	Protein	Attaches to the insulin receptor	Insulin-mimetic action	[60]
METRNL	Adipokine	Cause up regulation of the PPARγ pathway	Increase in the insulin sensitivity	[61] [62, 63]
PEDF (Pigment epithelium-derived factor)	Glycoprotein	Increase kinase-mediated Serine/Threonine phosphorylation cascade of IRS which causes insulin resistance	Decreasing level of PEDF increases the insulin sensitivity	[64]
VASPIN (SERPIN A12)	Serum glycoprotein	Vaspin performs its action by inhibiting the KLK7	Due to inhibition of KLK7, insulin signalling is improved and also the half-life of insulin is increased that helps in decreasing the blood glucose levels	[65, 66]
GPER (G protein-coupled estrogen receptor)	Glycoprotein	Regulation of glucose homeostasis by binding to both Gi/o and Gs proteins	Increase insulin secretion	[67–69]
GENE THERAPY	Gene	Act by correcting or repairing the defective genes	Suppression of auto reactive T cells to stop islet cells destruction	[70, 71]

#### 11β Hydroxysteroid dehydrogenase

**Fig. 9:**
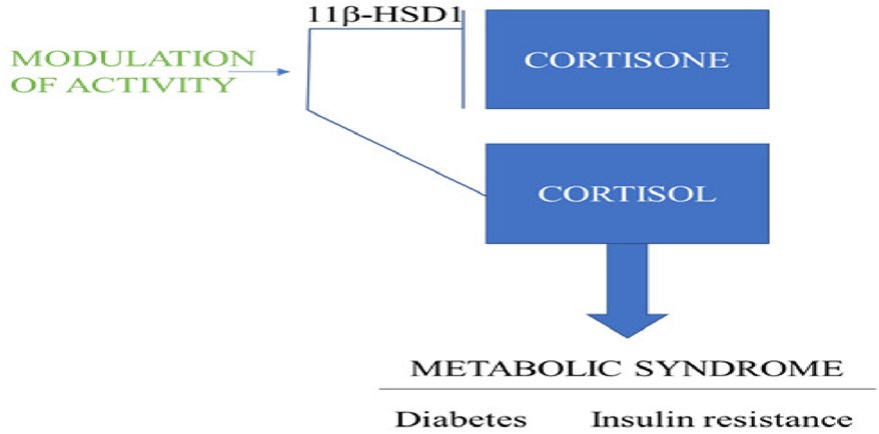
Working of 11β- HSD1

It is an enzyme that converts the cortisone which is a glucocorticoid [72] to its active form named cortisol [73]. It is currently available in these two isoforms which are 11-hydroxysteroid dehydrogenase type 1 (11β-HSD1) & 11β-hydroxysteroid dehydrogenase type 2 (11β-HSD2) [74]. It is stated that the high levels of glucocorticoid in the blood may cause glucose intolerance to the person, so by maintaining the levels of 11β-HSD1 enzyme it naturally improves insulin sensitivity [55]. Reported studies suggesting that in many diabetic and obese animal studies [75] or when the specific 11β-HSD1 knockout mouse is used then there is a decrease in blood glucose levels, improved insulin sensitivity [76], have a better glucose tolerance [77] and also the regeneration of glucocorticoids in the body was absent in them. So, it is concluded that inhibiting the 11β-HSD1 may work in reducing insulin resistance and thus increasing insulin sensitivity by regulating the insulin signaling transduction system (Fig. 9) [78]. By taking all into consideration presented above 11β-HSD1 is a novel molecular target for the treatment of diabetes mellitus.

#### ACRP-30

**Fig. 10:**
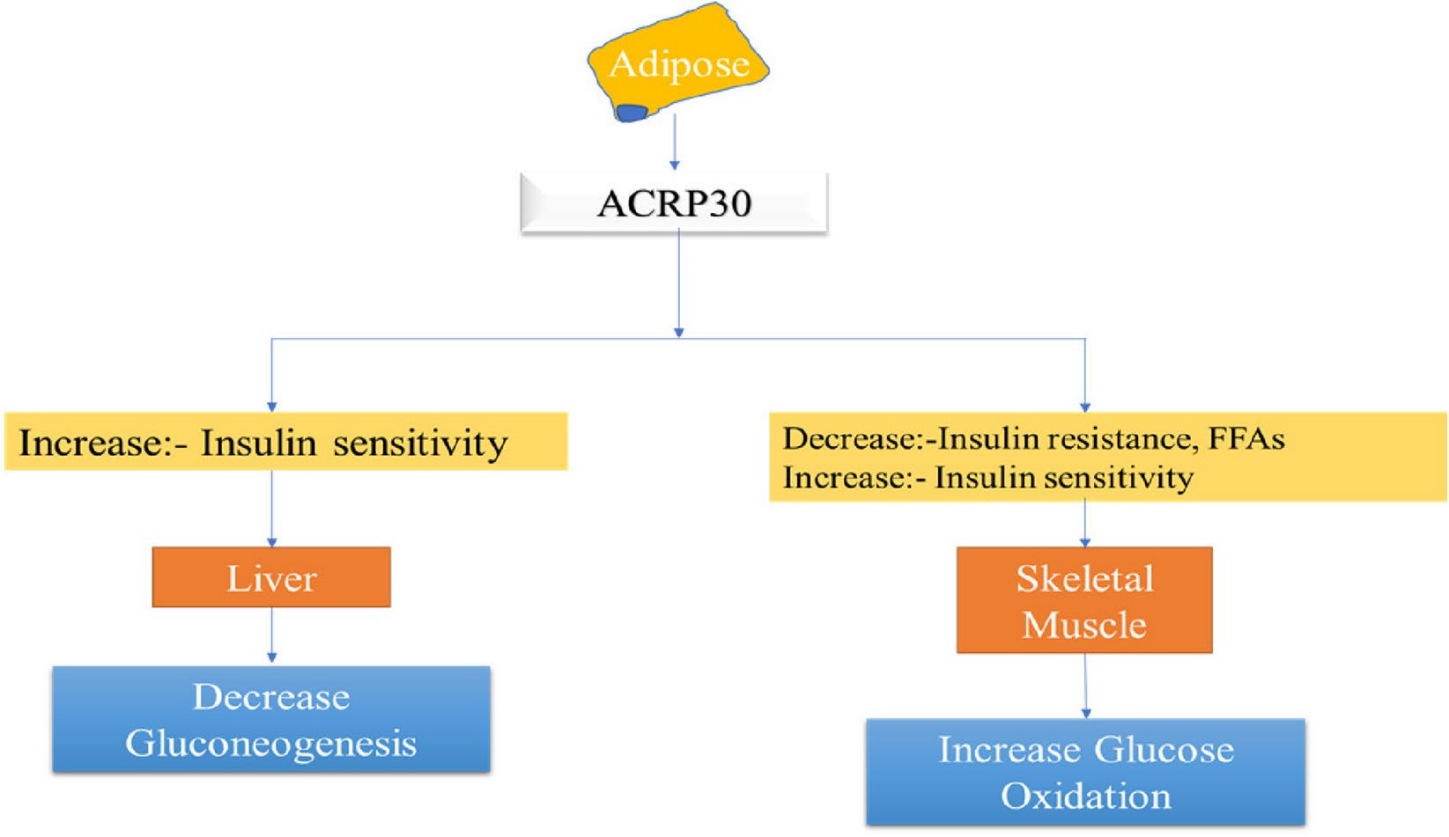
Role of ARCP30 in diabetes

Adipose tissue is long known for its ability to store fats but now the studies reveal that they also serve as a source of hormones like resistin, adipsin, leptin, TNF-α, adiponectin or Acrp30 [79]. It is discovered that serum protein Acrp30 performs a major part in the management of diabetes mellitus [79], TNF-α is one of the main pro-inflammatory mediators responsible for the insulin resistance [58]. It is also revealed from a study that Acrp30 levels are found to be decreased in many models of obesity and diabetes [80] due to high levels of TNF-α [56], which shows that this protein is negatively linked with diabetes (Fig. 10) [57], also showed that the mice lacking Acrp30 shows insulin resistance [58] leading to the development of diabetes mellitus. So, if the levels of Acrp30 will be increased in the circulation then the insulin sensitivity can be increased and blood glucose levels (Table 3) can be easily managed which will make Acrp30 a potential novel target for the treatment of diabetes mellitus.

**Table 3:** Functions of ACRP-30

ACRP-30 FUNCTIONS		
Actions	Target tissue	References
Reduces plasma glucose concentration	Entire body system	[81]
Improves insulin action	Liver	[81]
Upsurges fatty acid oxidation & reduces plasma fatty acid concentration	Skeletal muscle	[80]

#### FETUIN-A

**Fig. 11:**
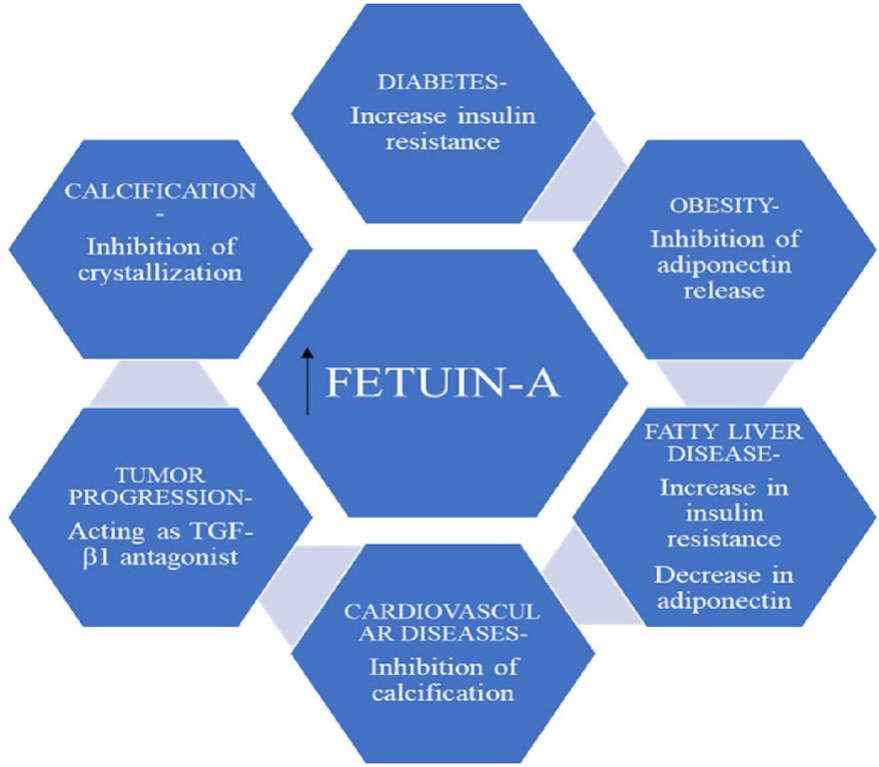
Role of Fetuin-A diabetes and other metabolic disorders

**Fig. 12:**
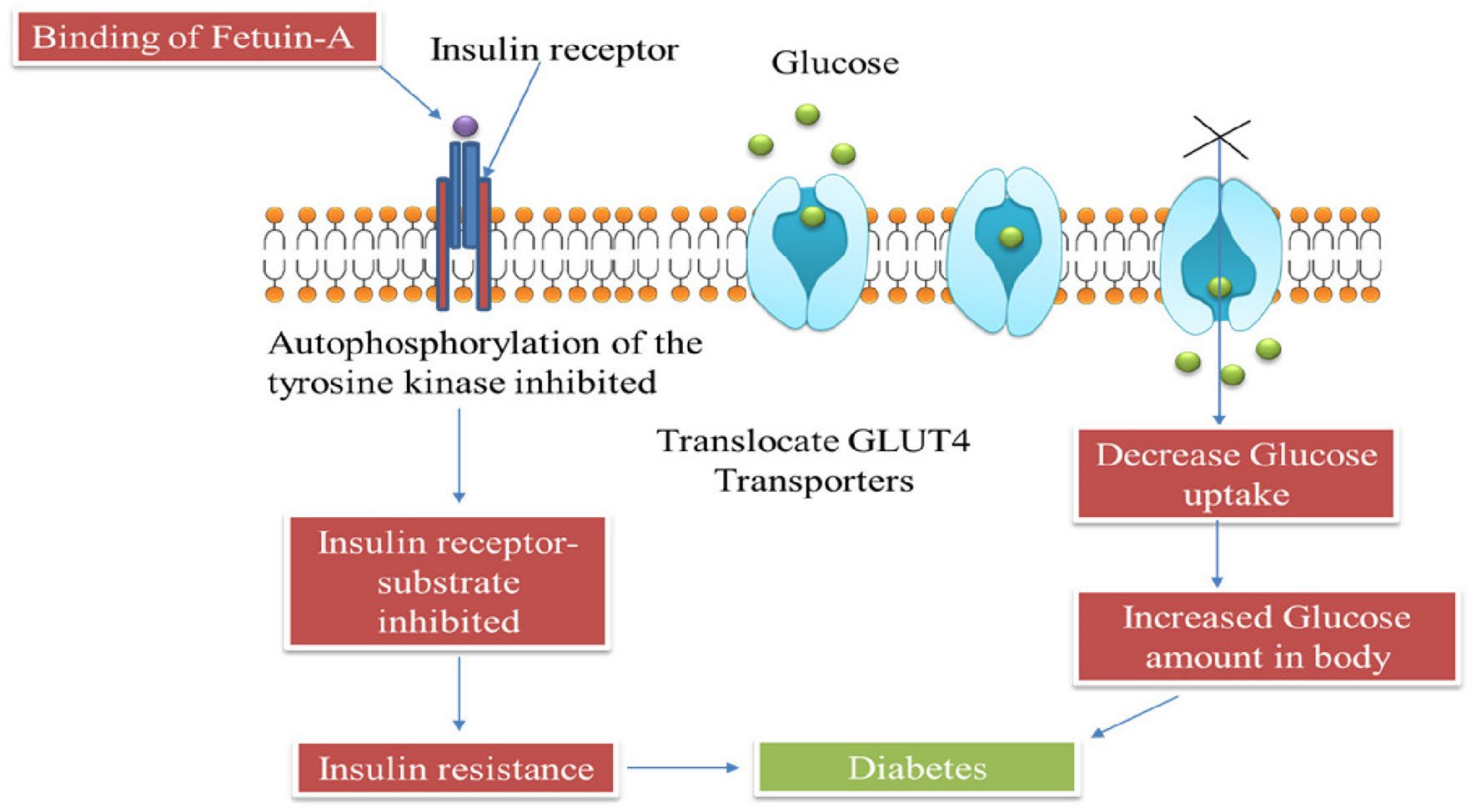
Mechanism of Fetuin-A in diabetes

It is a glycoprotein produced primarily from the liver & releases into the circulation [82]. Fetuin-A is the major protein required for carrying free fatty acids (FFA) to the circulation [83] and involved in the inflammation of the β-cells [59] and can leads to β-cell deterioration in the pancreas thus causing insulin resistance and some other metabolic disorders (Fig. 11). Along with the insulin, Fetuin-A is a major protein that can bind with the outer region of the insulin receptor [84]. Fetuin-A inhibit the autophosphorylation of the tyrosine kinase which is one of the main enzymes for the insulin signaling [85], that is totally opposite to the insulin action. There is the major interface among insulin and tyrosine kinase to balance the blood glucose in the system and if the concentration of the Fetuin-A will increase in the blood then the insulin resistance may occur in the body (Fig. 12) [59] and ultimately diabetes. Studies revealed that there is an increase in the insulin sensitivity in mice which are having Fetuin-A knockout genes in them [86] which shows the negative relation of the Fetuin-A with insulin sensitivity in diabetes [87]. These above listed factors indicate that Fetuin-A have potential to become a innovative aim for the management of diabetes mellitus in the future.

#### Visfatin

**Fig. 13:**
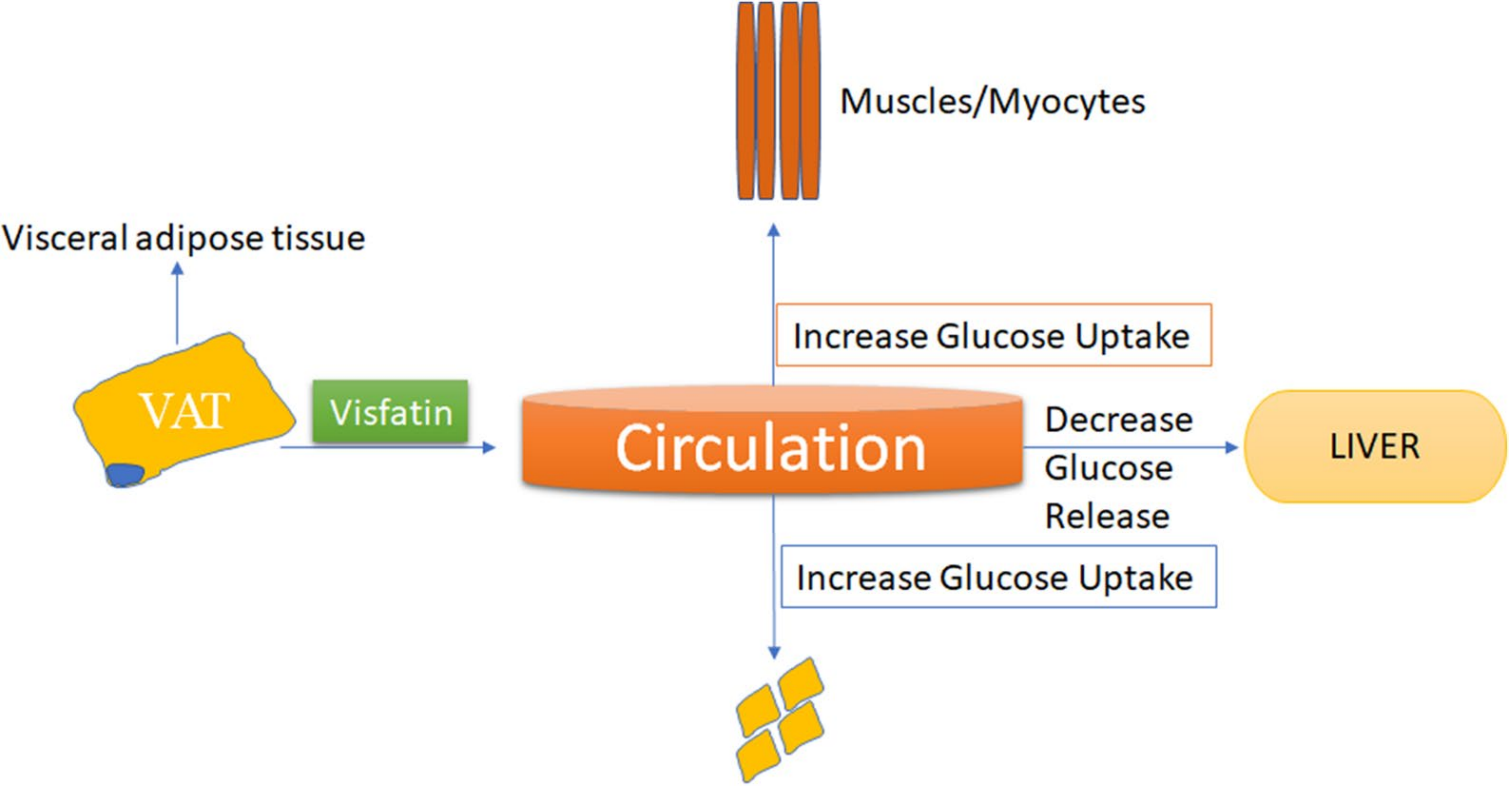
Role of visfatin in glucose homeostasis

Visfatin, a multifunctional protein also known as Nicotinamide phosphoribosyl-transferase [88] discovered in 2005 having different types (Table 4) [60]. It is found in number of tissues & organs like but mostly articulated in the visceral adipose tissue [60]. Previously it is also known as the PBEF(Pre-B colony Enhancing Factor) and has insulin-like features[89]which means it supports to recover insulin sensitivity[89]that indicates it may have a role in diabetes also and makes it a novel approach for the treatment of diabetes mellitus. It has been shown that the serum visfatin concentration are increased along-with the worsening of T2DM [90, 91] which creates a relation between visfatin and T2DM. Recent studies showed that visfatin attaches to the insulin receptor at a location other than that of insulin which shows that it has the insulin-mimetic action and enhances cell proliferation (Fig. 13) [60].

**Table 4:** Types of Visfatin

Types of Visfatin in different Human Tissues			
Tissue or cell	Type of Visfatin	Method of Determination	References
Subcutaneous adipose tissue	Visfatin mRNA	RT-PCR	[92]
Visceral adipose tissue	Visfatin mRNA	RT-PCR	[93]
Macrophages	Visfatin protein	Immunohistochemistry	[94]
3T3-L1 cell line	Visfatin mRNA	RT-PCR, Immunohistochemistry	[95]
Monocytes	Visfatin protein	Immunohistochemistry	[96]
Lymphocytes	Visfatin mRNA	RT-PCR	[97]
Skeletal muscle	Visfatin mRNA	RT-PCR	[92]
Placenta	Visfatin mRNA	RT-PCR	[89]
Fetal membranes	Visfatin protein	Northern blot	[98]
GI (colonic epithelium)	Visfatin mRNA	RT-PCR	[94]
Synovial fluid	Visfatin protein	ELISA	[99]
Plasma	Visfatin protein	ELISA, RIA	[100]

However, till now it is not clear how the Visfatin is completely related to diabetes but there are some stimulators and inhibitors of visfatin (Table 5), although scientists are working on the mechanism of visfatin in diabetes. With these evidences, it can be concluded that there is a correlation between diabetes and visfatin in the body which turns it into a possible target for the management of diabetes mellitus.

**Table 5:** Visfatin inhibitors and stimulators

Stimulators and inhibitors of Visfatin		
Stimulators	Inhibitors	Reference
Hypoxia, Hyperglycemia, Inflammation, TNF-alpha, IL-6, IL-1 beta, Chronic Kidney Disease, Labor/Pregnancy, PCOS(polycystic ovary syndrome), Cancer, HAART (highly active antiretroviral therapy), Spironolactone, CoCl_2_ (hypoxia mimetic agent) Macrostemonoside A	Insulin, Somatostatin, Monounsaturated fatty acid (e.g. oleate)	[89]

#### Metrnl

**Fig. 14:**
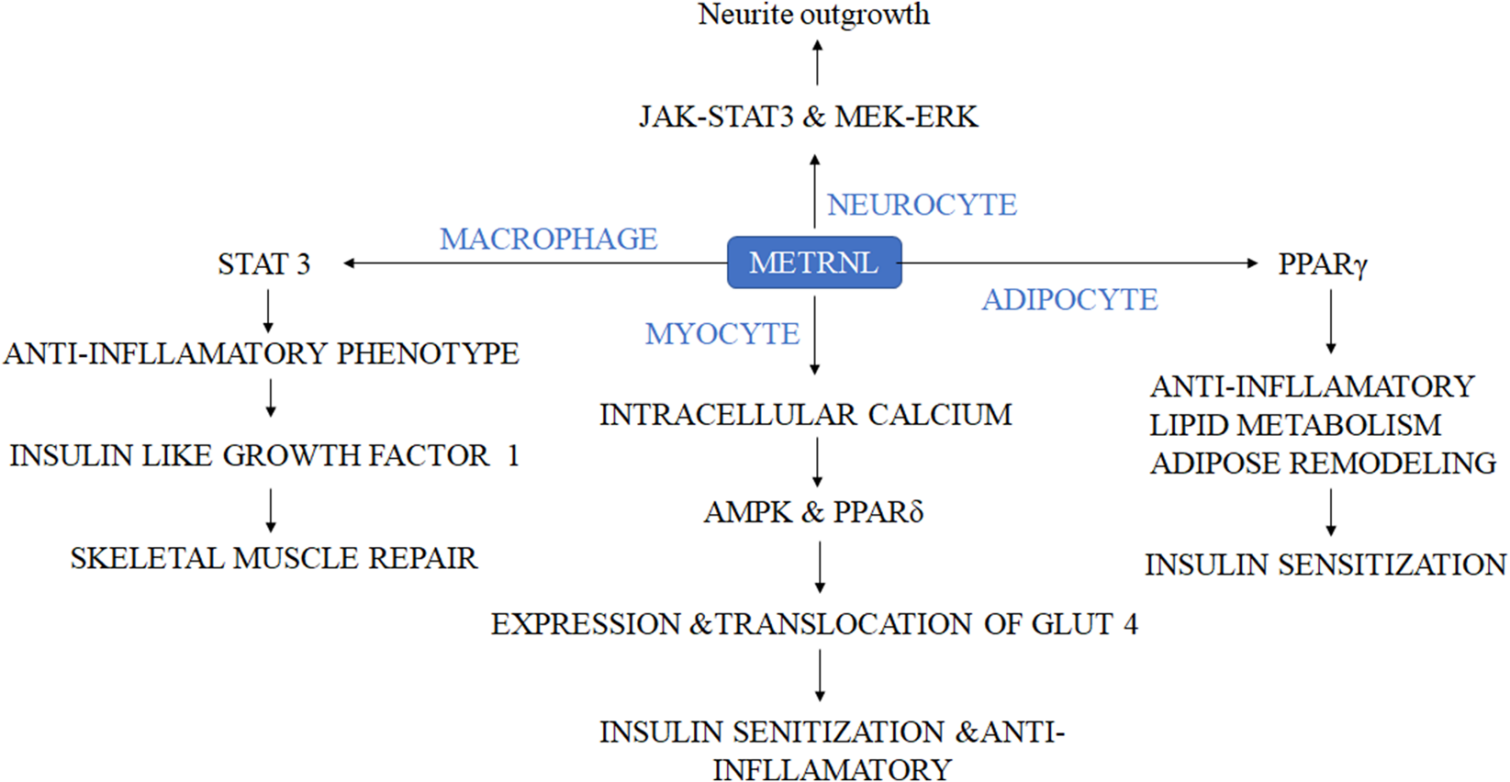
Metrnl is involved in various pharmacological pathways through intracellular signalling between the cells. In nerve cells, it promotes the neurite outgrowth via the JAKSTAT3 and MEK-ERK signalling pathway. In fat cells due to upregulation of the Metrnl increases the lipid metabolism, relieves from the high-fat diet-induced inflammation and improves adipose remodeling through upregulation of PPARγ, due to which the insulin resistance is also improved. In muscle cells or myocytes it increases the PPARγ signalling which increases the phosphorylation of AMPK due to increased intracellular calcium and also encourages the phosphorylation of TBC1D1, HDAC5, and p38 MAPK in an AMPK-mediated manner, then promotes the expression and translocation of GLUT4, which thus improves the insulin sensitivity and reduces the inflammation [103]

Metrnl is derived as an adipokine obtained from the adipose tissues which are abundantly present in the subcutaneous white fat in the body [101] which play an important role in maintaining glucose homeostasis (Table 6), Metrnl also plays a major role in maintaining energy metabolism, lipid metabolism, cardiovascular function, immunological inflammation and also in insulin sensitivity [62, 63]. In a study, researchers found that it works through the up regulation of the PPARγ pathway due to which there is an increase in the insulin sensitivity in Mice model [61]. Concurrently it is also found that, it promotes adipose tissue browning due to which there is an increase in energy expenditure and improved glucose tolerance (Fig. 14) [102].

**Table 6:** Clinical studies data of metrnl [103]

Sample size	Criteria	Results/ conclusion	References
139 subjects (47 subjects with normal glucose tolerance, 46 subjects with prediabetes, and 46 newly diagnosed T2D patients)	People with type 1 diabetes, gestational diabetes, active hepatitis/liver cirrhosis, chronic renal failure while on hemodialysis, congestive heart failure, or other major diseases were excluded	Lower serum Metrnl levels in subjects with newly diagnosed diabetes compared with those without diabetes	[104]
170 subjects (66 patients with CAD, 63 T2D patients and 41 controls)	Patients with > 70% stenosis in at least one coronary artery were diagnosed with CAD. Participants with history and evidence of stroke, myocardial infarction, etc. or using thiazolidinedione family drugs were excluded	Lower serum Metrnl in CAD and T2D patients compared to the control group	[105]
20 subjects (11 healthy controls, 9 patients with newly diagnosed T2D)	No other major diseases and treatment	Lower circulating Metrnl in people with newly diagnosed T2D	[106]
800 subjects (400 patients with T2D and 400 non-diabetes)	Over 40 years of age without a history of cardiovascular disease, without stage 2 hypertension, malignant disease, severe renal or hepatic disease	Blood Metrnl increased in patients with T2D	[107]
228 subjects (124 non-diabetes [73 non-obese and 51 obese] and 104 T2D [38 non-obese and 66 obese])	BMI > 30 kg/m^2^ were considered obese; Morbidly obese patients (BMI > 40 kg/m^2^) or subjects taking medication or supplements known to influence body composition or bone mass were excluded	Increased blood Metrnl in T2D and obesity	[108]
160 subjects (40 subjects with normal glucose tolerance, 40 subjects with impaired fasting glucose, 40 subjects with impaired glucose tolerance, and 40 patients newly diagnosed T2D)	Patients with previously diagnosed T2D, other types of diabetes, other major diseases, and medication history including the use of antidiabetics, statins, diuretics, corticosteroids, estrogen, and progestin were excluded	Increased Blood Metrnl in patients with T2D and significantly increased in patients with prediabetes compared with individuals with normal glucose tolerance	[109]
260 subjects (89 subjects with normal glucose tolerance, 77 subjects with glucose tolerance impairment and 94 with T2D)	BMI < 35 kg/m^2^; age between 20 and 75 years; no other CVD; no history of malignancy or recent infection; no history of taking antidiabetic medications, concomitant medications such as systemic steroids, cholestyramine, statins, diuretics, β-blockers, or oral anticoagulants	Decreased serum Metrnl level in patients with T2D versus subjects with normal glucose tolerance	[110]
89 subjects (59 T2D with durations ≥ 1 year and 30 healthy participants)	Patients with other types of diabetes and other major diseases were excluded	Increased blood Metrnl in patients with T2D. No relationship between Metrnl level and obesity-related indicators	[111]

#### PEDF (Pigment epithelium-derived factor)

**Fig. 15:**
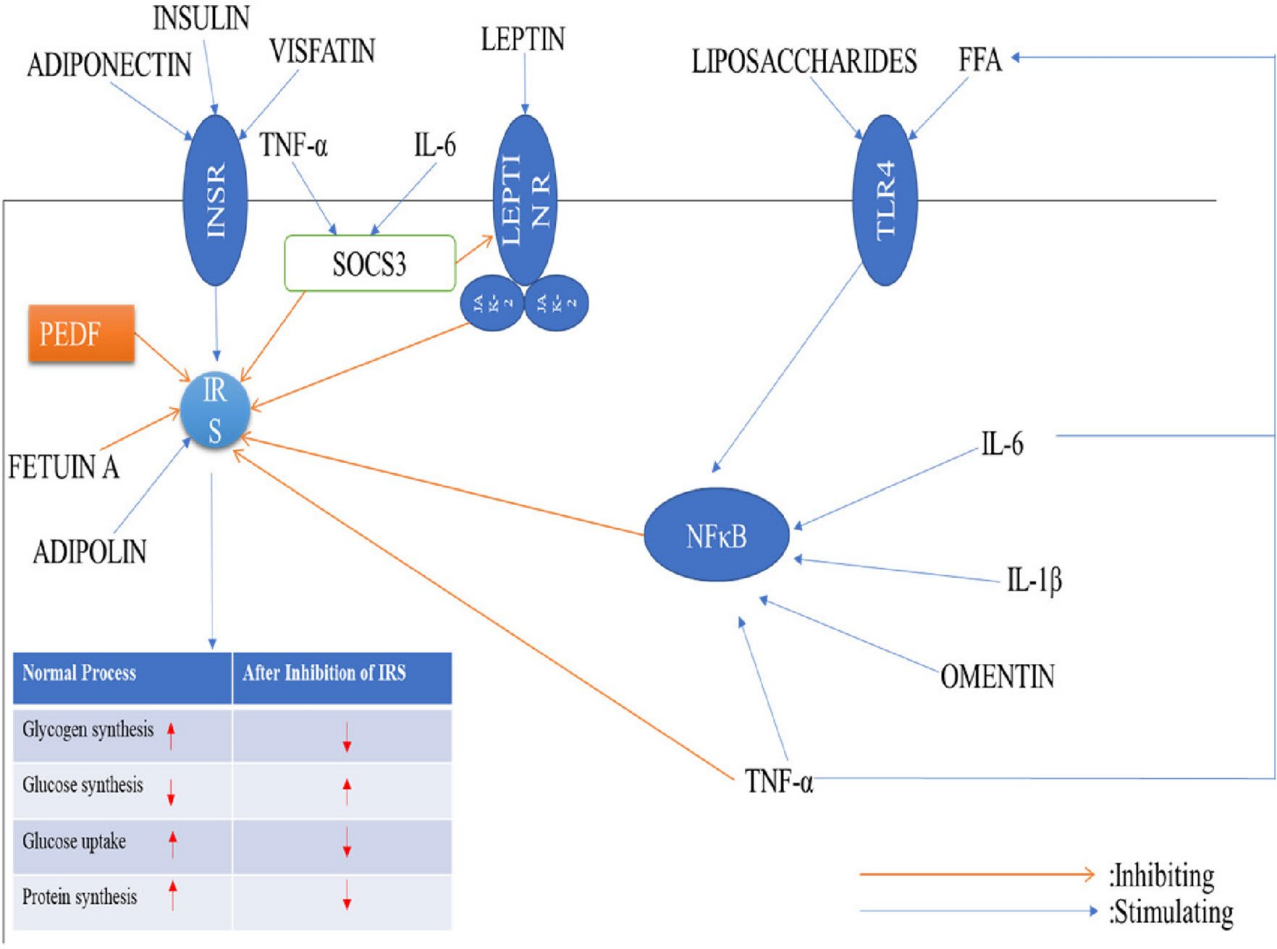
FFA: free fatty acids, INSR: Insulin receptor, IRS: insulin receptor substrate, JAK2: Janus kinase, LeptinR: Leptin receptor, NFκB: nuclear factor κ B, SOCS3: suppressor of cytokine signalling 3, TLR: toll-like receptor

It is a 50 kDa secreted glycoprotein released from the adipose tissue and human retinal pigment cells which belongs to the family of serine protease inhibitors [112]. It works in hydrolyzing the lipid triglycerides into glycerol and free fatty acids and thus moving the free fatty acids to the systemic circulation leading to inflammation in the cells. It gives rise to the kinase-mediated Serine/Threonine phosphorylation cascade of IRS (insulin receptor substrate), due to this process the insulin signaling is reduced which causes insulin resistance in the body cells [64]. Along with this, it also releases inflammatory mediators like TNF-α and IL-1(Interleukin-1) in the system due to that insulin insensitivity occurs in the body [112]. In a study it is investigated that after the administration of PEDF in animals there is a decrease in the insulin sensitivity which restores after the anti-PEDF given to them [113]. In children and adults, PEDF shows a positive correlation with insulin resistance [114]. So, if we can decrease the amount of PEDF in the circulation that may help to increase the insulin sensitivity, this makes PEDF a potential novel approach for the treatment of diabetes mellitus and other metabolic syndromes in the body. PEDF show its action by targeting the insulin receptor substrate (IRS) given in (Fig. 15) where it blocks the insulin signaling that further stops the process of glucose uptake by the cells, protein synthesis, glycogen synthesis which shows an increase in the amount of blood glucose levels in the body. The other factors which are also activated by the PEDF are the free fatty acid (FFA), toll-like receptor4(TLR4)**,** nuclear factor kappa B (NFκB), suppressor of cytokine signalling (SOCS3),Janus kinase (JAK2) which also blocks the insulin receptor substrate which together contributes in the decreased insulin sensitivity and ultimately diabetes mellitus (Fig. 15).

#### Vaspin (Serpin A12)

**Fig. 16:**
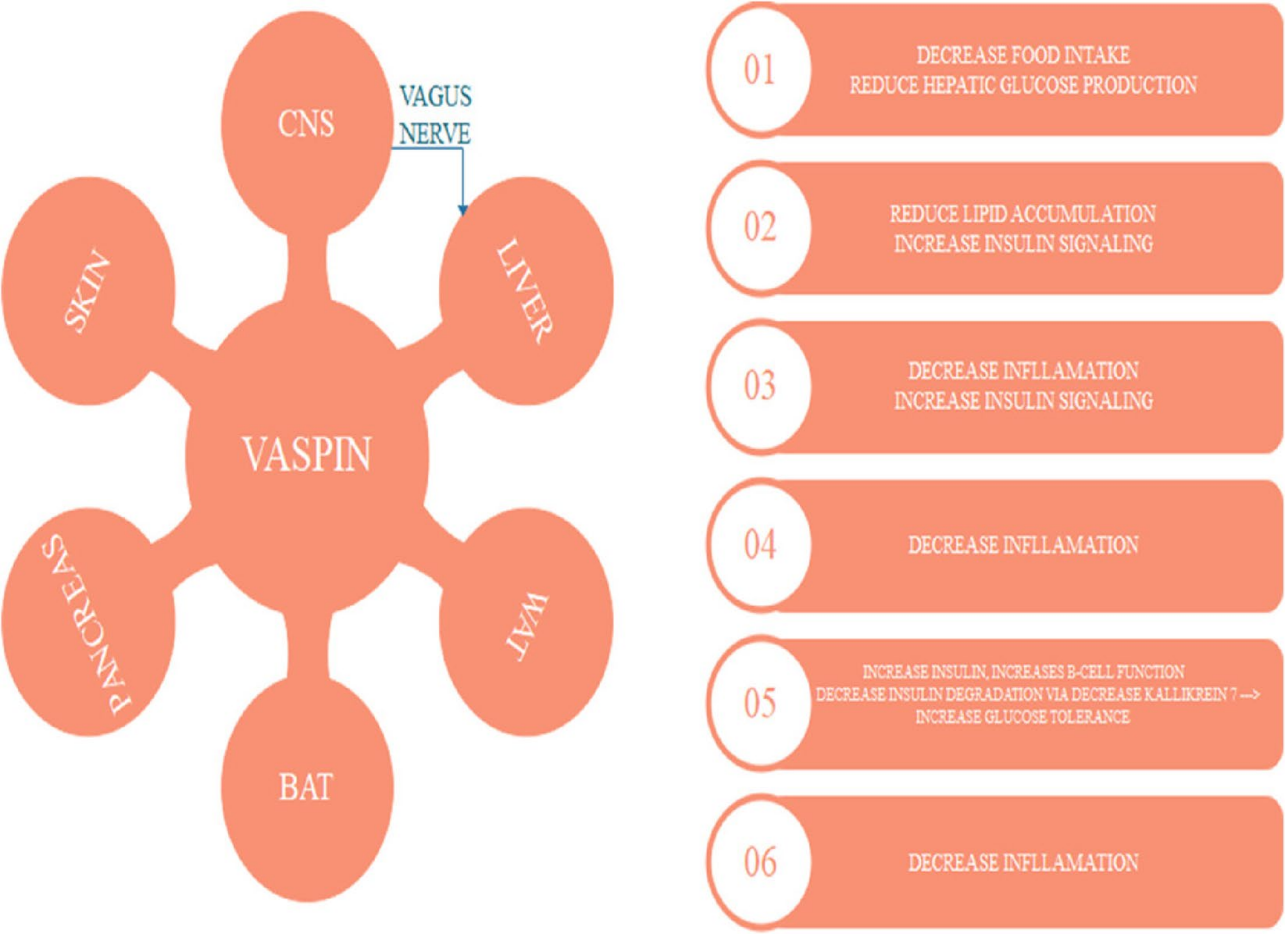
Role of vaspin in different organs linked to diabetes mellitus

Vaspin or Serpin A12 is a serum glycoprotein that comes under the serpin superfamily [115]. It is derived from the fat cells [116], plays an important role in modifying insulin activity [117]. It has been studied that, When diabetes severity increases, the serum levels of the vaspin start decreasing [118], this creates an idea that if the levels of vaspin start increasing in the circulation then it could be helpful in the management of diabetes mellitus. In animal studies, it has been also observed that the administration of the vaspin into the rats shows the improvement in insulin sensitivity along with increased glucose tolerance [116]. These evidences make it a potential target for the treatment of diabetes mellitus and other metabolic disorders like obesity (Fig. 16). Vaspin performs its action by inhibiting the KLK7 (kallikrein 7) which is an insulin-degrading enzyme that degrades the insulin and decreases the insulin half-life [65]. Due to the inhibition of KLK7, the insulin signalling is improved and also the half-life of insulin is increased that helps in decreasing the blood glucose levels [66]. It also performs some other actions which indirectly reduce the blood glucose from the body like it reduces the food intake that ultimately reduces the hepatic glucose production (HGP) via the hepatic branch of the vagus nerve by reducing hepatic lipid accumulation and increasing insulin signalling in the liver. In white adipose tissue (WAT) and Brown adipose tissue (BAT), it reduces inflammation and increases insulin signaling and in CNS central nervous system it decreases food intake by triggering the vagus nerve (Fig. 16) [119].

#### GPER (G protein-coupled estrogen receptor)

**Fig. 17:**
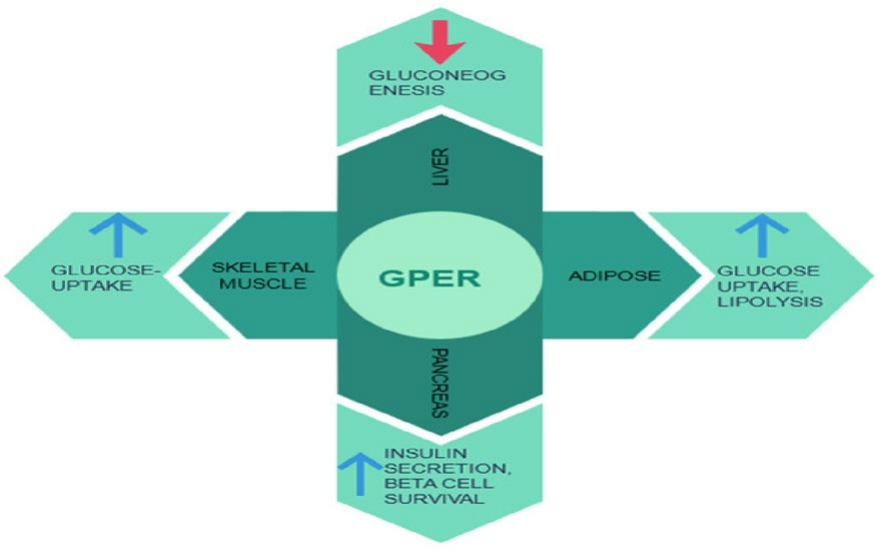
Role of GPER in different body organs affecting diabetes mellitus

GPER is an orphan 7-transmembrane G-protein coupled estrogen receptor [120, 121] that involved in estrogen signalling [122]. They are located in the intracellular membrane of cells [123] and plays an important role in the regulation of glucose homeostasis, inflammation [124], vascular tone and cell growth [122] by binding to both Gi/o and Gs proteins in the body [67]. In a GPER deficient female mouse model, it was found that there is an insufficient amount of insulin [68, 69] is producing in them that lead to the development of diabetes mellitus. It also has been shown in a study that in premenopausal women estrogen levels are high which shows the positive effects on maintaining the blood pressure, lipid metabolism, glucose homeostasis, as well as reducing inflammation [125] but after menopause when the estrogen levels start declining which makes the women more prone to the insulin resistance and multiple metabolic disorders all of them contributes to the diabetes mellitus [126]. These evidences suggest that GPER could play a crucial role in management diabetes and could become an interesting drug target for diabetes and related disorders (Fig. 17).

### Gene therapy

**Fig. 18:**
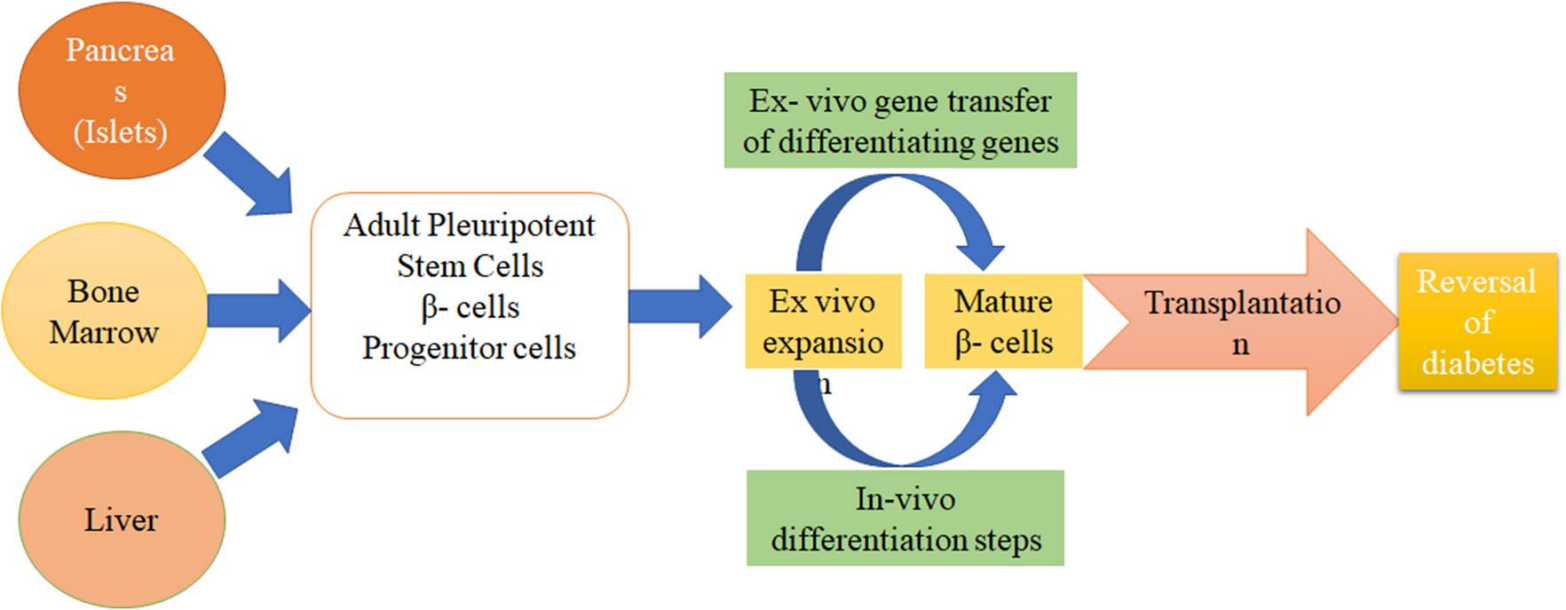
Systemic representation of the characteristics of cell-based gene therapy procedures in diabetes treatment [128]

Gene therapy is an emerging method for the treatment of diabetes mellitus that act by correcting or repairing the defective genes [71] which are responsible for diabetes mellitus. In this technique, transfer of genes can be done by the viral vector and non-viral transduction method to get the effect by the suppression of auto reactive T cells to stop islet cells destruction as a preventive method of treatment or the replacement of the insulin gene [70]. It is found in a study that the stem cells may be used for the treatment of diabetes serving as the surrogate β-cells [127] as they can multiply in the culture easily. It has also been studied that when the modified stem cells are transplanted into the mice by intrahepatic injection the level of blood glucose was found to be low (Fig. 18). When the mice are sacrificed for the histopathological studies, the distribution of stem cells shows green fluorescence under fluorescent microscope and insulin presence was identified by brown stain after staining with anti-human insulin polyclonal antibody. It showed that mesenchymal stem cells successfully expressed human insulin and was able to maintain normal blood glucose at the end of 42 days study [70] This was compared to the mice not treated by gene therapy. So, there is a scope in gene therapy as an evolving new technology that can used for the treatment of diabetes mellitus (Fig. 18).

## Conclusion and future perspectives

Diabetes is a worldwide epidemic and vulnerable disease from which large number of patient are suffering currently. The primary goal of every therapy in the treatment of diabetes mellitus is to attain near-normal blood glucose levels in the body. Treatments available for diabetes are only able to manage its symptoms and delay its progression but not able to cure it properly, along with there are also various side effects associated with their uses. Researchers are continuously working in search of new lead compounds for the proper cure for the diabetes mellitus and its complications and trying to make an approach in which the side effects should be minimal. The conventional approaches which has been used for a long time for the treatment of diabetes mellitus includes the Insulin therapy [5], Biguanides [8], Sulphonylureas [5], Glinides [5], Thiazolidinediones [5], Gliptins [5], α- Glucosidase inhibitors [5], Amylin analogues [5], SGLT-2 [9], Dopamine D-2 agonists [10]. As a primary targets they can only manage the symptoms and delay the progression and also consist of many side effects like weight gain, hypoglycemia, diarrhoea, nausea, mitogenic effect, bladder cancer etc., [5, 129, 130] which is not good for the patients who are dealing with these metabolic diseases. To overcome these side effects researchers are continuously searching for new targets for diabetic therapy, in last decade targets like PPAR’s are the primary focus of researchers but despite of enormous pre-clinical studies very few leads are in clinical studies and in market, Because of these facts we can’t rely upon the current approaches to diabetes treatment and should explore some new innovative pharmacological targets. In this view, receptor like GPCR 119 [13], GPER [14], 11β-hydroxysteroid dehydrogenase 1 [15], Vaspin [16], Metrnl [17], PEDF [18], Fetuin-A [19], ACRP 30 [20], Visfatin, Melatonin [21], GIP [22], GPCR [23] having direct or indirect role in insulin regulation as suggested by studies done. These receptors have potential to become targets in the treatment of diabetes and can become the landmark to find the permanent cure for diabetes and related complications. It is also suggested that in future there are possibilities in gene therapy or stem cells to become a therapeutic agent with better potential with lesser side effects.

## Data Availability

Not applicable.

## Abbreviations

ACRP: Adipocyte complement related protein
AMP: Adenosine monophosphate
AMPK: Adenosine monophosphate-activated protein kinase
BAT: Brown adipose tissue
BMI: Body mass index
BP: Blood pressure
cAMP: Cyclic adenosine monophosphate
CAD: Coronary artery disease
CCK: Cholecystokinin
CKD: Chronic kidney disease
CNS: Central nervous system
CVD: Cardiovascular disease
CVS: Cardiovascular system
DKA: Diabetic ketoacidosis
DPP: Dipeptidyl peptidase
ELISA: Enzyme-linked immunosorbent assay
EPAC: Exchange protein directly activated by cAMP
ERK: Extracellular-signal-regulated kinase
FFA: Free fatty acids
GIP: Glucose-dependent insulinotropic polypeptide
GLP: Glucagon-like peptide 1
GLUT: Glucose transporters
GPCR: G-Protein coupled receptors
GPER: G protein-coupled estrogen receptor
HAART: Highly active antiretroviral therapy
HDAC: Histone deacetylase
HDL: High-density lipoprotein
HGP: Hepatic glucose production
IL: Interleukin
INSR: Insulin receptor
IRS: Insulin receptor substrate
JAK: Janus activated kinase
JAKSTAT: Janus kinase-signal transducers and activators of transcription
KATP: ATP-sensitive potassium
KLK: Kallikrein 7
LCFAs: Long chain fatty acids
LDL: Low-density lipoprotein
WAT: White adipose tissue
UTI: Urinary tract infection
UKPDS: UK prospective diabetes study
TNF: Tumor necrosis factor
TLR: Toll-like receptor
STAT: Signal transducers and activators of transcription
SOCS: Suppressors of cytokine signaling
SGLT-2: Sodium-glucose cotransporter-2
SCFAs: Short-chain fatty acids
RNA: Ribonucleic acid
RIA: Radioimmunoassay
PYY: Peptide YY
PPAR: Peroxisome proliferator-activated receptor
PKA: Protein kinase-A
PEDF: Pigment epithelium derived factor
PCOS: Polycystic ovary syndrome
PBEF: Pre-B cell colony-enhancing factor
NPH: Neutral protamine Hagedorn
MT: Melatonin receptors
MEK: Mitogen-activated protein kinase
MCFAs: Medium-chain fatty acids
MAPK: Mitogen-activated protein kinase
